# Microbially Enhanced Oil Recovery by Alkylbenzene-Oxidizing Nitrate-Reducing Bacteria

**DOI:** 10.3389/fmicb.2019.01243

**Published:** 2019-06-18

**Authors:** Navreet Suri, Fatma Gassara, Paul Stanislav, Gerrit Voordouw

**Affiliations:** ^1^Petroleum Microbiology Research Group, Department of Biological Sciences, University of Calgary, Calgary, AB, Canada; ^2^Biopterre, Sainte-Anne-de-la-Pocatière, QC, Canada

**Keywords:** MEOR, alkylbenzenes, nitrate, *Thauera*, biomass, oil emulsification

## Abstract

Microbially enhanced oil recovery (MEOR) of heavy oil and bitumen is challenging because light hydrocarbons, which can feed resident microbial communities are present in low concentrations, if at all. We have recently shown that increasing the toluene concentration of heavy oil by aqueous injection followed by injection of nitrate boosts the activity of toluene-oxidizing nitrate-reducing bacteria in heavy oil-containing sand pack columns, giving production of residual oil in place (ROIP). In the current work we found that ethylbenzene is as effective as toluene. Microbial community analyses indicated *Thauera* and *Pseudomonas* to be main components of nitrate-containing batch and continuous cultures, regardless whether ethylbenzene or toluene was used as the electron donor. Biomass from batch cultures grown with heavy oil amended with ethylbenzene or toluene and nitrate or biomass from continuous cultures grown on ethylbenzene or toluene and nitrate had similar MEOR activity. Increasing the concentration of injected biomass from continuous cultures increased the fraction of ROIP recovered both in the absence and in the presence of nitrate. Nitrate increased the fraction of ROIP recovered by about 2-fold by increasing the concentration of biomass in the columns. Emulsification of oil by surface-adhering biomass and blocking of aqueous flow channels by oil emulsion droplets are proposed as a possible mechanism of hydrocarbon- and nitrate-mediated MEOR. Pure isolates *Thauera* sp. NS1 and *Pseudomonas* sp. NS2, which used both ethylbenzene and toluene, were obtained but did not offer improved MEOR compared to the use of batch and continuous cultures.

## Introduction

Alkylbenzenes, such as toluene, ethylbenzene and xylenes are monocyclic aromatic constituents of oil. Diverse alkylbenzene-oxidizing nitrate-reducing bacteria (ABO-NRB) of the phylum *Proteobacteria* have been enriched from hydrocarbon rich environments, including oil fields (Spormann and Widdel, 2000; Chakraborty and Coates, 2004; Lambo et al., 2008; Agrawal et al., 2012). This includes toluene-oxidizing NRB of the genera *Thauera* and *Azoarcus* (Rabus et al., 1999; Spormann and Widdel, 2000; Weelink et al., 2010). Enhanced growth of toluene-oxidizing NRB in heavy oil containing sand-packed columns increased recovery of residual oil in place (ROIP; Gassara et al., 2015). This was achieved through amendment or through sequential injection of aqueous toluene, to increase its concentration in oil, followed by injection of nitrate and NRB (Gassara et al., 2015). The formation of gaseous N_2_ and CO_2_, emulsifying biomass and/or biosurfactants were suggested as possible causes for this microbially enhanced oil recovery (MEOR).

Accumulation of biomass and associated exopolysaccharides can cause selective plugging of high permeability zones in bioreactors or reservoirs, which is one of the suggested mechanisms for MEOR (Sen, 2008; Youssef et al., 2009; Siegert et al., 2013). Increasing the toluene concentration in the oil phase and the nitrate concentration in the aqueous phase increased biomass growth and the proportions of *Thauera* and *Pseudomonas* species in the ABO-NRB consortia (Gassara et al., 2015; Suri et al., 2017). These taxa can produce exopolysaccharides that may also contribute to selective plugging (Allen et al., 2004; Schmid et al., 2015). In addition to toluene, *Thauera* and *Pseudomonas* species can also oxidize ethylbenzene under nitrate-reducing conditions (Chakraborty and Coates, 2004; Rabus, 2005; Weelink et al., 2010). Although toluene and ethylbenzene are chemically similar, the metabolic pathways for their oxidation are different (Champion et al., 1999), which may give changed metabolic outcomes (formation of biomass and biomolecules).

In the current paper the MEOR potential of using ethylbenzene was evaluated. Continuous cultures using ethylbenzene or toluene and nitrate were established and selected pure cultures of *Thauera* and *Pseudomonas* were isolated to identify the contribution of these taxa to hydrocarbon- and nitrate-mediated MEOR.

## Materials and Methods

### Batch and Continuous Cultures of ABO-NRB

Produced water from producing well 18PW in the Medicine Hat Glauconitic C (MHGC) field (Voordouw et al., 2009; Shen et al., 2018) from which heavy oil with an API gravity of 16° was produced was used as a source of microorganisms. For primary batch cultures 5 mL of 18PW was used to inoculate 44 mL of sterile anaerobic CSBK medium (Supplementary Table S1) either with or without 80 mM nitrate in 120 mL serum bottles. MHGC oil (1 mL) with or without additional ethylbenzene or toluene was added to these bottles (Table 1). The bottles were sealed with butyl rubber stoppers, crimped with aluminum seals and had a 70 mL headspace of 90% N_2_ and 10% CO_2_ (N_2_-CO_2_). These were incubated upside down on a horizontal shaker (80 rpm) at 30°C for 35 days. Secondary batch cultures in the same growth media as used for primary cultures were inoculated with 5 mL of centrifuged and re-suspended primary culture (Table 1: B_EN) as the inoculum. These were incubated with shaking for 20 days.

**Table 1:** The percentage (%) reduction of nitrate by ABO-NRB enriched in batch cultures containing 2% (v/v) MHGC oil with or without additional electron donor.

Designation	Source of ABO-NRB inoculum	Electron donor (mM) added to oil		Electron acceptor (mM)	Days of incubation	N^2^	Nitrate reduction^3^ (%)
		Ethylbenzene	Toluene	Nitrate			
B_N	18PW	0	0	80	35	3	2.4 ± 2.3
B_EN	18PW	475	0	80	35	4	20.2 ± 0.8
B_TN	18PW	0	570	80	35	3	41.8 ± 2.0
B_EN_EN	B_EN	475	0	90^1^	20	3	25.1 ± 1.4

*These were either inoculated with produced water 18PW from the MHGC field or ABO-NRB primary enrichment grown on 475 mM ethylbenzene in 1 mL of oil and 80 mM nitrate in 49 mL of aqueous phase (B_EN). Each value represents an average ± SD of three or four replicates. ^*1*^10 mM on day 0 and 80 mM on day 6. ^*2*^Number of replicates. ^*3*^Nitrate reduction (%) = (nitrite concentration/initial nitrate concentration)^∗^40% + ((initial nitrate concentration - residual nitrate concentration - nitrite concentration)/initial nitrate concentration)^∗^100%.*

Alkylbenzene-oxidizing nitrate-reducing bacteria continuous cultures were established by inoculating anaerobic CSBK medium with 10 mM nitrate and 10 mL of 18PW in 160 mL chemostats. Continuous cultures were either with or without 2 mL of heptamethylnonane (HMN) as the inert hydrocarbon carrier. To the HMN layer 60.6 mM ethylbenzene or 71.2 mM toluene was added for reduction of 10 mM nitrate in the 98 mL aqueous phase. When HMN was absent, 1.2 mM ethylbenzene or 1.4 mM toluene was dissolved directly in CSBK medium. Note that the solubilities of ethylbenzene and toluene in water at 20°C are 1.5 and 5.6 mM, respectively. The chemostats were sealed with butyl rubber stoppers, crimped with aluminum seals and had a 60 mL headspace of N_2_-CO_2_. Duplicate chemostats were incubated without flow on a horizontal stir plate (200 rpm) at 22–25°C for 9 days. The chemostats were then connected to influent from a medium reservoir and to effluent containers (Supplementary Figure S1) at an initial flow rate of 10 mL/day, corresponding to a dilution rate (D) of 0.1 d^-1^. Note that all the continuous culture chemostats received an input of CSBK medium with 10 mM nitrate and 1.2 mM ethylbenzene or 1.4 mM toluene. After 29 days of operation the flow rate was increased to 20 mL/day (0.2 d^-1^). Samples (200 μL) were collected periodically from the sampling port (Supplementary Figure S1) for nitrate and nitrite measurements using high performance liquid chromatography as explained earlier (Agrawal et al., 2012; Suri et al., 2017).

### Microbial Community Analysis of Batch and Continuous ABO-NRB Cultures

Primary and secondary batch cultures were transferred into 50 mL Falcon tubes at the end of incubation. While the continuous culture effluents (1 mL) were collected in 1.5 mL microcentrifuge tubes at the sampling port (Supplementary Figure S1) at different times of operation. Samples were centrifuged at 10,000 rpm for 30 min. Following centrifugation, the supernatants were carefully discarded and the cell pellets were used for extracting DNA. The extracted DNA was subjected to PCR amplification of 16S rRNA genes using primers based on 926Fw and 1392R in a two-step PCR procedure as described elsewhere (An et al., 2013; Suri et al., 2017; Menon and Voordouw, 2018). The resulting purified 420 bp PCR product was sequenced using the 300PE (paired-end) MiSeq protocol on an Illumina Miseq system in the Energy Bioengineering and Geomicrobiology Group, University of Calgary. The 300PE reads were merged using PEAR 0.9.6 with a minimum 50 bp overlap and were further processed with a 420 bp cutoff of amplicon size using MetaAmp, a 16S rRNA data analysis pipeline, which was also used for bioinformatic analysis^1^ (Dong et al., 2017). Quality controlled (QC) sequences were clustered into operational taxonomic units (OTU’s) using average neighbor clustering at a distance of 3%. OTU’s were assigned to taxa by comparing with the latest version of the non-redundant 16S rRNA small subunit SILVA database. Samples were clustered into a dendrogram using the unweighted pair group method algorithm (UPGMA) and the distance between communities was calculated as the Bray-Curtis coefficient in Mothur software (Dong et al., 2017). The dendrogram was visualized using the Mega 7.0.26 (Kumar et al., 2016). The entire sets of 16S rRNA gene amplicon raw reads have been submitted to the NCBI sequence read archive (SRA) under Bioproject accession numbers SAMN10280305 to SAMN10280325.

### Isolation and Identification of ABO-NRB

Serially diluted samples from continuous cultures were plated on CSBK medium with 10 mM acetate and 10 mM nitrate, solidified with 15 g/L of agar. The plates were incubated upside down at room temperature (22–25°C) in an anaerobic hood with an atmosphere of N_2_-CO_2_. Individual colonies were picked and grown in 1 mL of anaerobic CSBK medium with 10 mM acetate and 10 mM nitrate in 2 mL screw capped glass vials. After 3 days of incubation, 100 μL of cultures that showed high turbidity were transferred to 1 mL of CSBK medium amended with 10 mM nitrate and with 5 mM benzoate, 1.2 mM ethylbenzene or 1.4 mM toluene. Transferred cultures that grew with all three of the electron donors were streaked on CSBK medium with 10 mM nitrate, solidified with 15 g/L of agar. The plates were incubated at room temperature (22–25°C) in anaerobic jars containing a crystallizing dish with 200 μL of ethylbenzene or toluene in 2 mL of HMN. The jars were kept in the anaerobic hood throughout the incubation. Colonies picked from these plates were suspended in 10 μL Tris-EDTA buffer (Sigma-Aldrich) in 200 μL PCR tubes (Bio-Rad) and incubated for 5 min at 95°C. 16S rRNA genes were then amplified with primers 27F and 1525R (Frank et al., 2008) in a 50 μL volume containing premade PCR reagents (Thermo Fisher Scientific). The PCR was for 25 cycles of 30 s at 95°C, 2 min at 72°C, followed by a final incubation at 72°C for 7 min. The quality of the PCR products was evaluated by electrophoresis on a 1.5% (w/v) agarose gel. PCR products were purified using the QIAquick PCR purification kit (Qiagen, Germany), quantified and subjected to Sanger sequencing at the Core DNA Services Laboratory of the University of Calgary^2^. The resulting 1500 bp sequences were compared with sequences in the GenBank database of the National Center for Biotechnology Information (NCBI) using the nucleotide-nucleotide blast (blastn) network service^3^. The 16S rRNA gene sequences of *Thauera* sp. NS1 and *Pseudomonas* sp. NS2 isolated in this study were submitted to GenBank with accession numbers MK085068 and MK085069, respectively.

### Phylogenetic Analysis of 16S rRNA Gene Sequences of *Thauera* Species

The 16S rRNA gene sequences of different *Thauera* species obtained from the NCBI nucleotide database^4^ are listed in Supplementary Table S2. *Thauera* affiliated 16S rRNA sequences from batch and continuous cultures were obtained with MetaAmp (see text footnote 1; Dong et al., 2017). These sequences were aligned using ClustalX version 2.0 in the multiple alignment mode (Larkin et al., 2007). The aligned sequences were trimmed to 420 bp by removing the upstream and downstream flanking regions including the primer sequences using BioEdit Sequence Alignment Editor version 7.2.5 (Hall, 1999). Phylogenetic analysis of the trimmed aligned sequences was carried out using MEGA version 7.0.26 (Kumar et al., 2016). The 16S rRNA sequence of *Acetobacter peroxydans* (GenBank Accession no. AB032352) was used as an outgroup in the phylogenetic analysis. Distances were computed using distance options according to Kimura’s two-parameter model (Kimura, 1980) and clustering was performed with the neighbor-joining method (Saitou and Nei, 1987). Statistical support for branches of the phylogenetic tree was obtained through bootstrap analysis based on 1500 resamplings (Felsenstein, 1985).

### Growth of ABO-NRB Isolates on Different Electron Donors

Isolated ABO-NRB were grown in 120 mL serum bottles with sterile anaerobic CSBK medium with 10 mM nitrate. Higher nitrate concentrations were used in some experiments. The bottles had 1 mL of HMN (2% v/v of total volume of 50 mL). Either 6.5 mM of aqueous acetate, 1.7 mM of aqueous benzoate, 60.6 mM of ethylbenzene in HMN or 71.2 mM of toluene in HMN was added as electron donor. Samples (200 μL) were taken periodically into 1.5 mL microfuge tubes using N_2_-CO_2_ flushed syringes to measure nitrate and nitrite concentrations by high performance liquid chromatography.

### Oil Production From Up-Flow Model High-Pressure Columns

Vertically oriented stainless steel sand-pack columns were prepared and operated at high pressure (27.2 atm) and at room temperature (22–25°C). Their pore volumes (PVs) were calculated by weighing them dry and following saturation with CSBK under upflow conditions. The columns were then injected with 1 PV of MHGC oil, which was unamended or amended with 9.5 mM ethylbenzene or 11.2 mM toluene. The injected heavy oil replaced approximately 0.95 PV of CSBK medium from the columns. Heavy oil was then produced from the columns by injection of CSBK at a rate of 1 PV/day for 2 weeks. Produced oil was measured by dichloromethane extraction of the oil-water effluent mixture followed by determination of the absorbance at 600 nm (A_600_) of the oil-dichloromethane layer as described previously (Kryachko and Voordouw, 2014; Gassara et al., 2015). This allowed calculation of the volume (mL) of residual oil in place (ROIP). Approximately 0.5 PV of ROIP remained in the columns. The columns were then injected with ABO-NRB in 0.5 PV of CSBK with or without 80 mM nitrate. Following injection, columns were closed at both ends and incubated under pressure without flow for 2.5 weeks (Gassara et al., 2015). Upflow injection of CSBK medium was then resumed at a rate of 1 PV/day. Apart from measurement of oil produced from the columns, the effluents collected were also analyzed for the concentrations of nitrate and nitrite in the aqueous phase using HPLC and of ethylbenzene or toluene in the oil phase using GC-MS as described elsewhere (Agrawal et al., 2012; Gassara et al., 2015; Suri et al., 2017).

## Results

### Nitrate Reduction by ABO-NRB in Batch Cultures

The concentrations of ethylbenzene and toluene in MHGC oil as determined by GC-MS analysis were 3.0 ± 0.9 and 2.6 ± 0.6 mM, respectively. Incubation of CSBK medium with 80 mM nitrate and 1 mL of MHGC oil inoculated with 18PW gave reduction of 2.4 ± 2.3% of nitrate after 35 days (Table 1: B_N). Adding 475 mM ethylbenzene or 570 mM toluene to the 1 mL oil phase, sufficient for reduction of 80 mM nitrate in the 49 mL aqueous phase, increased the reduction of nitrate to 20.2 ± 0.8 and 41.8 ± 2.0%, respectively (Table 1: B_EN and B_TN). Secondary batch culture incubation of primary enrichment B_EN in medium with nitrate and MHGC oil with 475 mM ethylbenzene gave 25.1 ± 1.4 % nitrate reduction (Table 1: B_EN_EN). Hence, significantly increased nitrate reduction was obtained by adding ethylbenzene or toluene to the oil phase.

### Nitrate Reduction by ABO-NRB in Continuous Cultures

**FIGURE 1:**
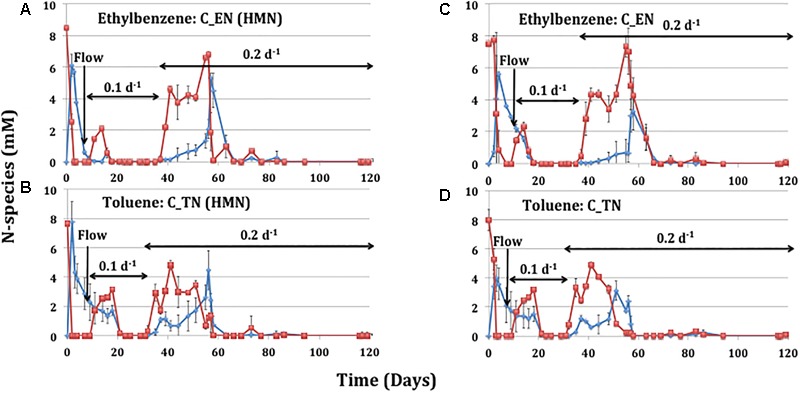
Concentrations of nitrate (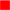) and nitrite (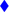) in effluents of ABO-NRB continuous cultures as a function of time. The continuous cultures were maintained at dilution rates of 0.1 or 0.2 d^-1^. Prior to starting flow the cultures were grown in 98 mL of aqueous medium with 10 mM nitrate and **(A)** 60.6 mM ethylbenzene in 2 mL of HMN, **(B)** 71.2 mM toluene in 2 mL of HMN or in 100 mL of aqueous medium with 10 mM nitrate and **(C)** 1.2 mM of aqueous ethylbenzene or **(D)** 1.4 mM of aqueous toluene. During flow the continuous cultures received medium with 10 mM nitrate and 1.2 mM of aqueous ethylbenzene **(A,C)** or 1.4 mM of aqueous toluene **(B,D)**.

Batch cultures of ABO-NRB reduced 80 mM nitrate only partially in the presence of heavy oil with added ethylbenzene or toluene (Table 1). To try to achieve more complete nitrate reduction and thus potentially higher biomass concentrations continuous cultures of ABO-NRB were started. ABO-NRB were initially enriched by inoculation of the CSBK medium in the chemostats with 18PW and incubation in the absence of flow (Figure 1: day 0 to 9). Nitrate (10 mM) was completely reduced to nitrite after 3 and 2 days of incubation in cultures with 60.6 mM ethylbenzene and 71.2 mM toluene in the HMN phase, respectively (Figure 1A,B). The reduction of nitrate to nitrite in cultures with 1.2 mM ethylbenzene and 1.4 mM toluene in the aqueous phase without HMN was slower with complete reduction of nitrate to nitrite observed after 7 and 3 days, respectively (Figure 1C,D). The formation of nitrite in all these cultures was transient with most being reduced to N_2_ at day 9. Flow was then started by injection of CSBK medium with 10 mM nitrate and 1.2 mM ethylbenzene or 1.4 mM toluene at a rate of 10 mL/day (dilution rate, *D* = 0.1 d^-1^). The effluent nitrate concentrations increased to 2–3 mM in the chemostats with an HMN phase (Figure 1A,B) and without an HMN phase (Figure 1C,D). However, the effluent concentrations of nitrate and nitrite decreased to zero at day 20 in all continuous cultures, irrespective of the presence or absence of an HMN phase (Figure 1). Increasing the rate of medium influx to 20 mL/day (*D* = 0.2 d^-1^) gave incomplete reduction of nitrate and nitrite with effluent concentrations of up to 5 to 7 mM from day 35 to 65 (Figure 1) after which the effluent nitrate and nitrite concentrations remained at zero. Overall, the nitrate and nitrite concentrations in the chemostat effluents increased transiently with each increase in dilution rate. These increases were higher in the transition from *D* = 0.1 d^-1^ to *D* = 0.2 d^-1^ than in the transition from *D* = 0 d^-1^ to *D* = 0.1 d^-1^ (Figure 1). There was little difference in the kinetic profiles of nitrate reduction and nitrite production and reduction between chemostats fed with ethylbenzene or toluene and between chemostats with or without an HMN phase. The biomass concentrations in the ethylbenzene-fed and the toluene-fed continuous cultures, as measured by the OD_600_, at 65 days were similar at OD_600_ = 3.12 and OD_600_ = 3.35, respectively.

### Microbial Community Compositions of Produced Water and of Batch and Continuous ABO-NRB Cultures

**FIGURE 2:**
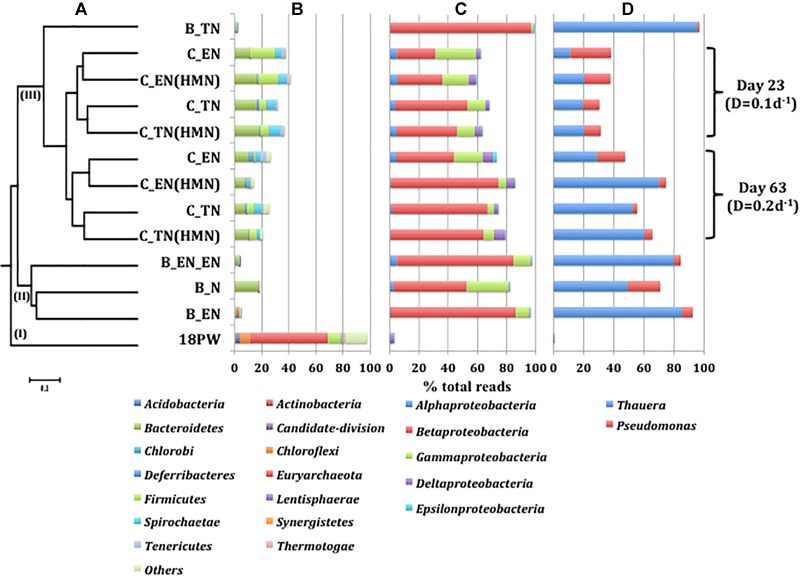
**(A)** Relational tree of microbial community compositions derived from Illumina sequencing of 16S rRNA amplicons of produced water 18PW and of batch and continuous cultures of ABO-NRB described in Table 1 and Figure 1. The scale indicates 10% sequence divergence. The distribution is shown of **(B)** phyla excluding *Proteobacteria*, **(C)** classes of the phylum *Proteobacteria* and **(D)** the genera *Thauera* and *Pseudomonas* in the microbial community compositions.

The microbial community compositions of produced water 18PW, batch cultures and continuous cultures are compared in Figure 2 and in Supplementary Table S3. A dendrogram of these compositions indicated that 18PW was in a separate clade I. Clade II contained most of the batch culture community compositions, whereas clade III contained the microbial communities of batch culture B_TN and of all of the continuous cultures. Within clade III, the microbial communities of continuous cultures at day 23 (*D* = 0.1 d^-1^) and at day 63 (*D* = 0.2 d^-1^) formed two subclades. Further subclustering separated microbial communities of ethylbenzene-fed from those of toluene-fed cultures. The presence or absence of an HMN phase had the smallest impact on microbial community compositions (Figure 2). The microbial communities within a particular clade had a similar composition at the phylum level (Figure 2B), had a similar distribution of classes within the phylum *Proteobacteria* (Figure 2C), and had similar proportions of the genera *Thauera* and *Pseudomonas* (Figure 2D).

The microbial community of the 18PW produced water sample, which was produced together with MHGC oil, was more diverse than the communities in batch cultures with MHGC oil with additional ethylbenzene or toluene and nitrate, as indicated by Shannon indices of 2.3 and of 0.5–1.6, respectively. The microbial community compositions of continuous cultures at day 23 (*D* = 0.1 d^-1^) had higher Shannon indices of 2.8–2.9 than those at day 63 (*D* = 0.2 d^-1^), which were 1.5–2.6 (Supplementary Table S3). The presence of HMN decreased the diversity at *D* = 0.2 d^-1^, but not at *D* = 0.1 d^-1^. The presence of HMN decreases the aqueous alkylbenzene concentration available to the microbial community, whereas a decrease in dilution rate decreases the specific growth rate constant of the microbial community.

Produced water 18PW had a high proportion of the phylum *Euryarchaeota* (Figure 2B), genus *Methanoculleu*s (Supplementary Table S3: 53%). This taxon was a minor community component in batch cultures with nitrate and heavy oil with or without additional ethylbenzene or toluene and was absent from continuous cultures with nitrate and ethylbenzene or toluene. These were, instead, dominated by the phylum *Proteobacteria* (Figure 2C) with the genus *Thauera* being most prominent throughout (Figure 2D and Supplementary Table S3). The genus *Pseudomonas* was on average the second most prominent taxon. The fraction of *Thauera* was highest in batch culture B_TN (95.3%). This batch culture also had the highest percentage of nitrate reduction (Table 1). Batch cultures B_EN and B_EN_EN also had high fractions of *Thauera* of 85.3 and 79.8%, respectively.

The average fraction of *Thauera* in continuous cultures was 17.8% at day 23 (*D* = 0.1 d^-1^) and increased to 52.9% at day 63 (*D* = 0.2 d^-1^). This increase in the fraction of *Thauera* was seen irrespective whether ethylbenzene or toluene was the electron donor and was stronger in continuous cultures with an HMN phase as compared to the cultures without an HMN phase. The average fraction of *Pseudomonas* in continuous cultures was 16.6% at day 23 (*D* = 0.1 d^-1^), which decreased to 7.8% at day 63 (*D* = 0.2 d^-1^). These results indicated *Thauera* to be the main player in catalyzing alkylbenzene oxidation coupled to nitrate reduction. The increased diversity of continuous cultures as compared to batch cultures was associated with higher fractions of phyla other than *Proteobacteria* (Figure 2B) and of genera *Acetoanaerobium, Desulfuromonas*, and *Sphaerochaeta* (Supplementary Table S3).

### Identification of Isolated ABO-NRB

Colonies with different sizes and morphologies appeared on agar surfaces plated with continuous ABO-NRB cultures. Two distinct bacterial isolates were obtained by subsequent plating of individual colonies picked from these plates. Both were able to grow in the presence of acetate, benzoate, ethylbenzene or toluene as electron donors and nitrate as the electron acceptor. The full length 16S rRNA sequence of isolate NS1 had 99.0% 16S rRNA sequence identity to *Thauera aromatica* strain S100 (Mechichi et al., 2002), whereas that of isolate NS2 had 99.0% 16S rRNA sequence identity to *Pseudomonas stutzeri* strain NBRC 12695 (Nakagawa et al., 2011). The *Pseudomonas* sp. NS2 colonies were bigger and were disc shaped with ridges radiating from the center, whereas the *Thauera* sp. NS1 colonies were smaller with a smooth surface appearance (Supplementary Figure S2).

### Comparison of Isolated *Thauera* Species With Those in ABO-NRB Cultures

**FIGURE 3:**
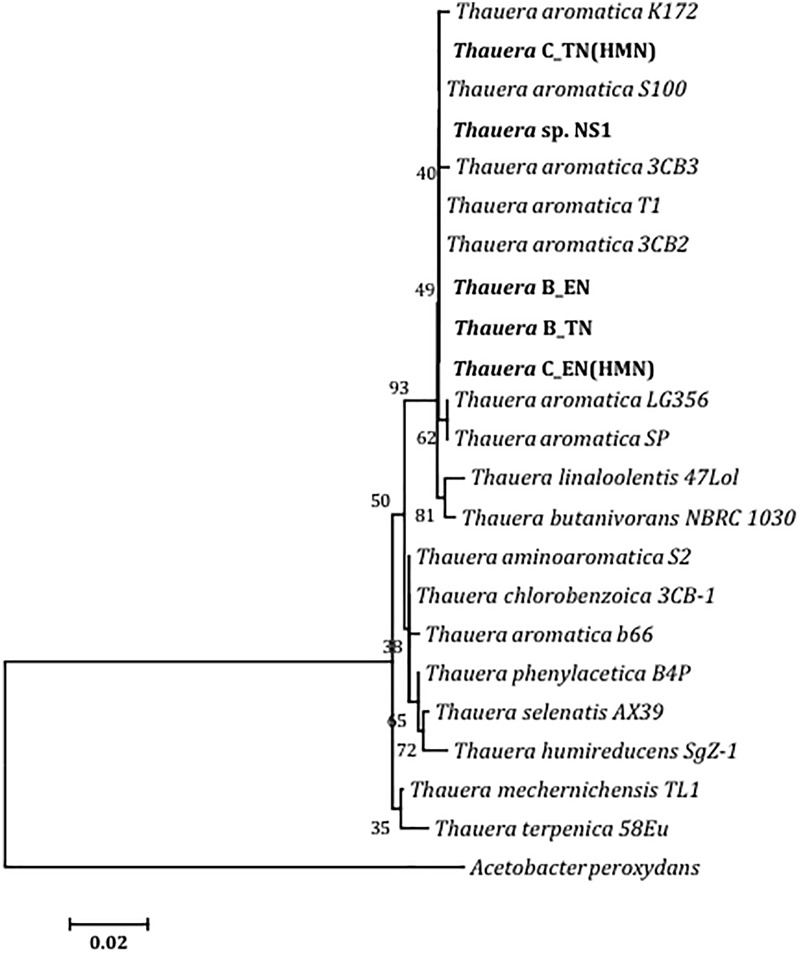
Neighbor-joining phylogenetic tree for a 420 nt region of 16S rRNA gene sequences. These were obtained by amplification with primers derived from 926Fw and 1392R of *Thauera* species in microbial communities of batch and continuous cultures and of the colony purified *Thauera* sp. NS1 and are compared with known sequences for species of *Thauera* (Supplementary Table S2). Numbers at branching points refer to bootstrap values (% of 1500 resamplings). The scale bar is 2 nucleotide substitutions in 100 nt.

A comparative phylogenetic analysis of the 420 bp region of the 16S rRNA gene sequences of *Thauera* species in microbial communities of ABO-NRB batch and continuous cultures and of *Thauera* sp. NS1 with sequences retrieved from the NCBI database (Supplementary Table S2) showed their similarity to *Thauera aromatica* (Figure 3). The 420 bp 16S rRNA sequences of *Thauera* sp. B_TN, B_EN, C_TN(HMN) and C_EN(HMN) from batch or continuous cultures and of the isolated *Thauera* sp. NS1 were found to be very similar to those of *T. aromatica* strains T1, 3CB2, 3CB3, S100, and K172 (Figure 3 and Supplementary Table S4: 98.3–100% identity). These sequences were less similar to those of other *Thauera* species (Figure 3 and Supplementary Table S4: 97.6–99.0% identity).

### Nitrate Reduction by ABO-NRB Isolates in Batch Cultures

**FIGURE 4:**
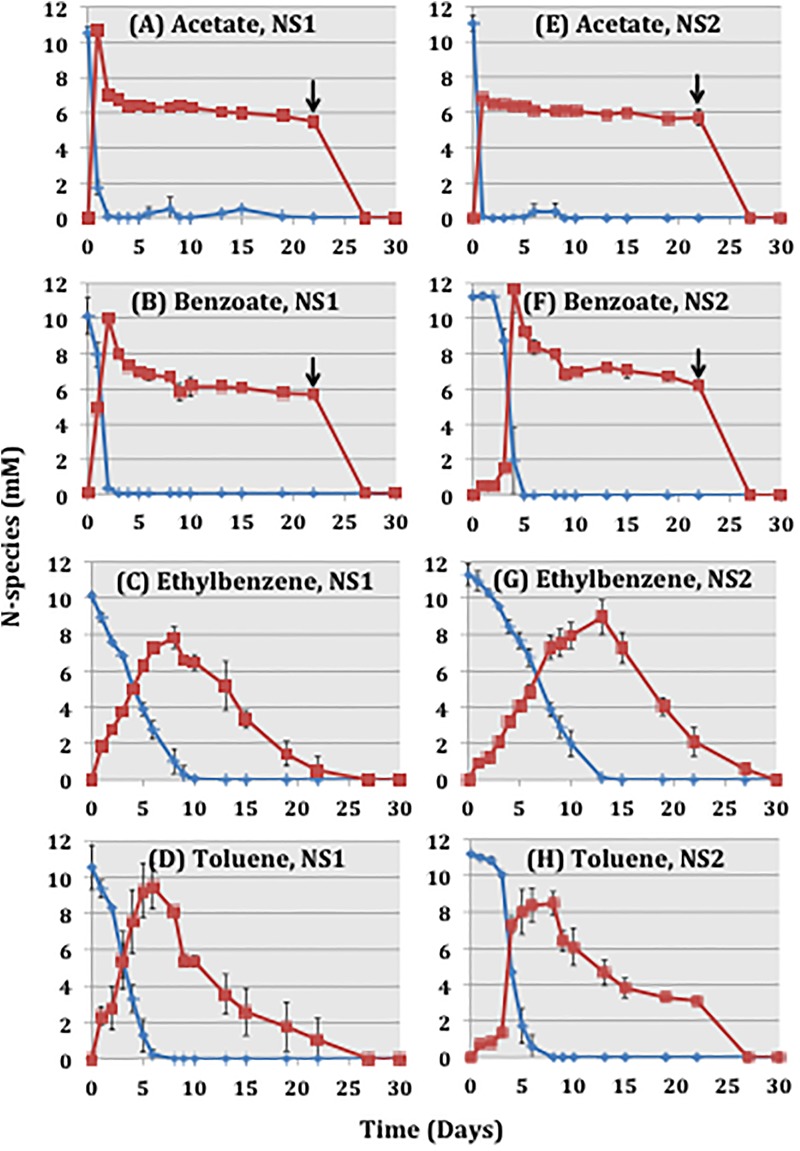
Concentrations of nitrate (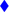) and nitrite (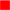) in batch cultures with *Thauera* sp. NS1 **(A–D)** and *Pseudomonas* sp. NS2 **(E–H)** with 6.5 mM acetate **(A,E)**, 1.7 mM benzoate **(B,F)**, 60.6 mM ethylbenzene in 1 mL of HMN **(C,G)** or 71.2 mM toluene in 1 mL of HMN **(D,H)** as electron donors and 10 mM nitrate as electron acceptor in 49 mL of aqueous phase. An additional 5 mM acetate **(A,E)** or 5 mM benzoate **(B,F)** was added to the incubations at the indicated times (↓).

Nitrate (10 mM) was completely reduced to N_2_ with transient formation of nitrite in batch cultures with *Thauera* sp. NS1 or *Pseudomonas* sp. NS2 or both (Figure 4 and Supplementary Figure S3). Based on the equations presented in Supplementary Table S5 reduction of 10 mM nitrate is coupled to the oxidation of 6.25 mM acetate, 1.72 mM benzoate, 1.39 mM toluene, or 1.19 mM ethylbenzene. Adding these stoichiometric concentrations gave complete reduction of nitrate to nitrite and of nitrite to N_2_ with ethylbenzene and toluene (Figure 4C,D,G,H) but not with acetate and benzoate, where approximately 6 mM nitrite remained (Figure 4A,B,E,F). This indicates formation of larger amounts of other products, e.g., biomass or exopolysaccharides (Chen et al., 2017). Addition of another 5 mM acetate or benzoate completed the reduction of nitrate. The kinetics of nitrate reduction was similar for the two isolates. NS2 oxidized benzoate, ethylbenzene and toluene somewhat more slowly than NS1. Incubations with equal proportions of NS1 and NS2 gave nitrate reduction rates similar to those of *Thauera* sp. NS1 (Supplementary Figure S3).

Batch cultures of *Thauera* sp. NS1 or *Pseudomonas* sp. NS2 with excess ethylbenzene or toluene as electron donor and 20, 40 or 80 mM nitrate as electron acceptor gave nearly complete reduction of nitrate to N_2_ (98.0 ± 1.8%) in cultures with 20 mM nitrate. Batch cultures with 40 or 80 mM nitrate reduced on average 40.3 ± 11.4% and 29.0 ± 12.9 % of nitrate, respectively, after 30 days of incubation (Table 2). The percent nitrate reduction observed with ethylbenzene was higher than with toluene in these incubations. Incubations with equal proportions of the two isolates did not significantly change the percentage of nitrate reduction (Table 2). Comparing the results obtained for batch cultures of NS1 and/or NS2 with those for enrichment cultures of 18PW indicated that none of these were able to reduce 80 mM nitrate with excess ethylbenzene or toluene in the HMN or in the oil phase, respectively (Tables 1, 2).

**Table 2:** The percentage (%) reduction of nitrate by *Thauera* sp. NS1 and *Pseudomonas* sp. NS2 in batch cultures containing 2% (v/v) HMN phase with dissolved electron donors.

ABO-NRB isolate inoculum	Electron donor (mM)		Electron acceptor (mM)	Nitrate reduction^1^ (%)
	Ethylbenzene	Toluene	Nitrate	
NS1	475	0	20	95.3 ± 1.1
NS1	0	570	20	98.4 ± 2.0
NS2	475	0	20	98.3 ± 1.8
NS2	0	570	20	97.1 ± 1.6
NS1+NS2	475	0	20	99.4 ± 0.1
NS1+NS2	0	570	20	99.4 ± 0.2
**Average ± SD**				**98.0 ± 1.8**
NS1	475	0	40	33.9 ± 6.9
NS1	0	570	40	31.8 ± 0.6
NS2	475	0	40	56.3 ± 2.7
NS2	0	570	40	33.9 ± 2.8
NS1+NS2	475	0	40	54.5 ± 0.5
NS1+NS2	0	570	40	31.7 ± 1.8
**Average ± SD**				**40.3 ± 11.4**
NS1	475	0	80	27.7 ± 4.1
NS1	0	570	80	10.1 ± 6.1
NS2	475	0	80	37.5 ± 2.5
NS2	0	570	80	33.9 ± 2.8
NS1+NS2	475	0	80	38.4 ± 0.9
NS1+NS2	0	570	80	30.2 ± 0.5
**Average ± SD**				**29.0 ± 12.9**

*Each value represents an average ± SD of two replicates. ^*1*^Nitrate reduction (%) = (nitrite concentration/initial nitrate concentration)^∗^40% + ((initial nitrate concentration - residual nitrate concentration - nitrite concentration)/initial nitrate concentration)^∗^100.*

### Oil Production From Heavy-Oil Containing Columns at High Pressure

Heavy oil-containing columns at high pressure (27.2 atm) were used to determine the effect of ABO-NRB on oil production, because these offer a better simulation of the reservoir environment than low pressure (1 atm) columns (Gassara et al., 2015). Less gas phase N_2_ and CO_2_, which displaces the residual oil in place (ROIP), is produced at higher pressure. Flooding of the columns with CSBK medium allowed the pore volume (PV) to be measured (Supplementary Table S6). Columns were then flooded with 1 PV of MHGC oil, usually with an added concentration of 9.5 mM ethylbenzene or 11.2 mM toluene then again with 15 PV of CSBK over a 15-day period. By monitoring the accumulative oil production, the ROIP could be estimated (Supplementary Table S6). The fraction of the pore volume occupied by oil (ROIP/PV) at the start of the MEOR experiment varied from 0.34 to 0.62 (Table 3) with the average being 0.49 ± 0.08 (*N* = 23). Injection of 0.5 PV of an ABO-NRB consortium with or without 80 mM nitrate was then done, as indicated in Table 3.

**Table 3:** Summary of oil production from high pressure columns.

Column	ROIP/PV^1^	Substrates^2^	ABO-NRB^3^ inoculum	ABO-NRB inoculum (OD_600_)	Oil produced (% ROIP)^4^
1, 2	0.53 ± 0.02	T	None	0	2.5 ± 0.1
3, 4	0.42 ± 0.01	N	None	0	5.4 ± 4.5
5, 6	0.39 ± 0.01	EN	B_EN	ND^5^	16.0 ± 1.9
7	0.58	E	C_EN (HMN)	0.090	6.9
8	0.62	E	C_EN (HMN)	0.092	5.5
9	0.47	T	C_TN (HMN)	0.819	11.1
10	0.43	T	C_TN (HMN)	1.769	14.2
11	0.60	EN	C_EN (HMN)	0.096	9.4
12	0.54	EN	C_EN (HMN)	0.105	9.2
13	0.47	TN	C_TN (HMN)	0.634	16.0
14	0.42	TN	C_TN (HMN)	1.238	21.9
15–17	0.48 ± 0.03	TN	NS1	0.281	10.5 ± 4.5
18–20	0.51 ± 0.03	TN	NS2	0.251	7.3 ± 1.9
21–23	0.51 ± 0.05	TN	NS1 + NS2	0.242	10.3 ± 5.3

*These contained approximately 0.5 PV of heavy MHGC oil and 0.5 PV of aqueous phase. Ethylbenzene (E) or toluene (T) were added to the oil phase, whereas nitrate (N) and ABO-NRB inoculum were injected with the aqueous phase, as indicated. Following 2.5 weeks of incubation flow was resumed and oil production was measured. Additional information is provided in Supplementary Table S6.^*1*^ROIP (mL) = PV (mL) – produced oil (mL). ^*2*^Substrates were (E) 9.5 mM of ethylbenzene in the oil phase, (T) 11.2 mM toluene in the oil phase, and/or (N) 80 mM nitrate in the aqueous phase of the columns. ^*3*^Inocula as in Table 1 and Figure 1, 3. ^*4*^(%) ROIP = [Oil produced (mL)/ROIP (mL)]^∗^100. ^*5*^ND, not determined.*

The negative controls (columns 1 to 4) lacked injected ABO-NRB and also either alkylbenzene or nitrate. These gave production of 2.5 ± 0.1% and 5.4 ± 4.5% of ROIP, respectively (Table 3). An average value of 4.1 ± 2.6% (*N* = 4) was therefore used as the value for the negative control.

Columns 5 and 6, containing heavy oil spiked with 9.5 mM ethylbenzene and injected with ABO-NRB and 80 mM nitrate, gave 16.0 ± 1.9% of ROIP (Table 3). This value was similar as obtained for heavy oil spiked with 11.2 mM toluene in previous work under the same conditions (Gassara et al., 2015), which was 17.9 ± 1.7% (*N* = 3) of ROIP. Nitrate was reduced in both of these sets of columns (Supplementary Table S6: 89.3 ± 1.0 and Gassara et al. (2015): 80.5 ± 7.8%, respectively). A larger increase in differential pressure (ΔP) was observed in columns with nitrate than in columns without nitrate, when water injection was restarted after incubation with ABO-NRB (Supplementary Figure S4). This was also observed for columns with oil with added toluene (Gassara et al., 2015). In terms of the Darcy equation the increased ΔP was interpreted as being required to compensate for a decrease in the effective area A for water flow through the columns following incubation (Gassara et al., 2015). The decrease in A may reflect blocking of aqueous flow channels through produced biomass and/or emulsified oil droplets.

Hence, increasing the concentration of ethylbenzene or toluene in heavy MHGC oil allowed reduction of more nitrate and gave a net production of 12.0 to 13.9% of ROIP. Ethylbenzene was as effective as toluene.

### Oil Production as a Function of ABO-NRB Biomass Concentration

Continuous cultures growing on ethylbenzene or toluene and nitrate were used to determine the effect of injected biomass concentration on the production of oil. The biomass concentration, determined as the OD_600_, was 3.12 and 3.35 for continuous cultures at 65 days grown on ethylbenzene or toluene, respectively. OD_600_ cannot be used as a proxy for the biomass concentration in batch cultures grown in the presence of oil, which were injected in columns 5 and 6 (Table 3). A volume of 10 mL of the continuous cultures was centrifuged and re-suspended in 10 mL of CSBK and further diluted with CSBK to OD_600_ values ranging from 0.090 to 1.769 (Table 3). These diluted ABO-NRB cultures contained either 0 or 80 mM nitrate and were then injected into columns 7 to 14, containing oil with ethylbenzene or toluene. On average 57 to 96% of the injected nitrate was reduced during the subsequent 2-week incubation. Upon completion of incubation and resumption of CSBK injection these columns produced 5.5 to 21.9% of ROIP (Table 3).

**FIGURE 5:**
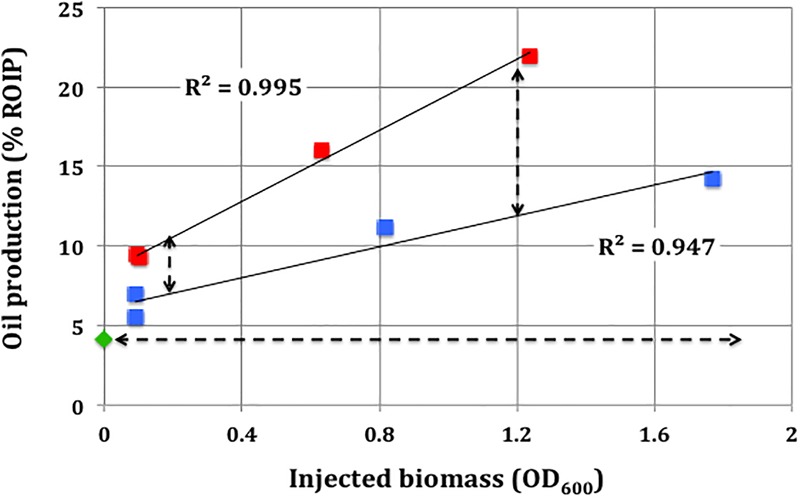
Oil production from high pressure columns as a function of injected biomass concentration (OD_600_). Oil production (% ROIP) from columns incubated with injected biomass without nitrate (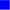) and with 80 mM nitrate (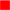) is shown. The diamond (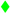) represents a negative control without added inoculum. The horizontal dotted line indicates oil production in the negative control. The vertical dotted lines indicate the increase in oil production due to the presence of nitrate.

Oil produced (% ROIP) increased with the OD_600_ of injected biomass both in the absence and in the presence of nitrate, as indicated by equations 1 and 2, respectively, which were obtained by linear regression of the data in Figure 5.

$$\begin{array}{l} \%\text{ROIP}\;\text{=}\;\text{4}.{\text{8OD}}_{\text{600}}\text{+6}.\text{1}(r^{\text{2}}\;\text{=}\;\text{0}.\text{947};N\;\text{=}\;\text{4})\text{ Eq}.\text{1}\\ \%\text{ROIP}\;\text{=}\;\text{11}.{\text{2OD}}_{\text{600}}\text{+8}.\text{3}(r^{\text{2}}\;\text{=}\;\text{0}.\text{995};N\;\text{=}\;\text{4})\text{ Eq}.\text{2} \end{array}$$

Hence, at a given biomass concentration the presence of nitrate increased oil production (% ROIP) by about 2-fold (Figure 5). A given production of additional oil (% ROIP) required an injected biomass concentration that was 3.3-fold lower in the presence than in the absence of nitrate. This likely reflected the growth of ABO-NRB in the presence of nitrate. Nitrate reduction was coupled to the oxidation of ethylbenzene or toluene in the oil phases of the columns. The average residual concentrations of ethylbenzene and toluene were 0.2 ± 0.3 mM (*N* = 2) and 0.9 ± 0.8 mM (*N* = 2), respectively, in oil produced from columns with nitrate, whereas in columns without nitrate 6 to 7 mM of ethylbenzene or toluene remained in the oil produced after incubation.

### Oil Production Using ABO-NRB Isolates

Isolates NS1 and NS2 were grown in batch cultures in medium with 10 mM acetate and 10 mM nitrate. The grown cultures were centrifuged and the cells were resuspended either separately or together in CSBK medium with 80 mM nitrate. High pressure columns 15 to 23, containing residual heavy oil with 11.2 mM of toluene, were injected with resuspended cultures, which had an OD_600_ of 0.258 ± 0.020. Following incubation and flooding with CSBK medium, these gave similar oil production. Columns inoculated with *Thauera* sp. NS1, *Pseudomonas* sp. NS2 or with both strains produced 10.5 ± 4.5, 7.3 ± 1.9, or 10.3 ± 5.3% ROIP, respectively (Table 3). The average nitrate reduction observed in the aqueous phase of the effluents from these columns was 56.6 ± 8.9% (Table 3: *N* = 9). Oil production calculated based on equation 2 obtained for continuous culture ABO-NRB consortia for an OD_600_ = 0.258 is 11.2% of ROIP. The values obtained for pure culture isolates thus appear slightly lower and these do not offer an MEOR advantage compared to continuous cultures.

## Discussion

Microbially enhanced oil recovery often involves injection of (i) cheap carbohydrate (e.g., molasses) as a fermentation substrate, (ii) microbes that catalyze the desired fermentation outcome, (iii) limiting nutrients (e.g., phosphate) and (iv) an electron acceptor like nitrate. Fermentation of injected carbohydrate can yield organic acids, alcohols, gasses, biosurfactants, biomass and associated polymers such as exopolysaccharide (EPS) (Sen, 2008; Youssef et al., 2009; Siegert et al., 2013), which contribute to MEOR in various ways. For instance, Youssef et al. (2007) showed that injection of two pre-cultured, lipopolypeptide biosurfactant-producing *Bacillus* strains, together with glucose, nitrate and salts increased oil production. The production of this lipopolypeptide biosurfactant in the oil-bearing subsurface, decreasing the oil-water interfacial tension, was demonstrated and was held responsible for the production of additional oil.

Production of biomass and associated EPS or other polymers can contribute to the plugging of high permeability zones in bioreactors or reservoirs, which is another suggested mechanism for MEOR. Because injected carbohydrate, nutrients, nitrate and microbial inoculum move primarily into high permeability zones, these will be blocked preferentially by microbial growth and associated polymer production (Youssef et al., 2009). Formation of EPS or other biopolymers augments the volume of the biomass. e.g., high density bacterial biomass occupies only about 0.4% of the volume of the aqueous phase (Supplementary Figure S5), but this can be enhanced considerably by biopolymer formation. Only polymer-producing bacteria have, therefore, been used successfully to stimulate oil recovery from sandpacks, carbonate cores and oil wells by flow diversion (Youssef et al., 2009).

Disadvantages of molasses-based MEOR is that the technology does not use the hydrocarbon substrate available downhole and that all substrates and fermenting microorganisms must be injected simultaneously. Reaction starts as soon as all component are mixed together and depth of penetration is thus dependent on injection flow rate versus reaction half life. In contrast, in hydrocarbon- and nitrate mediated MEOR oil hydrocarbons like toluene are used as substrates. However, because these are often in short supply in heavy oils, MEOR is improved by increasing the concentration of toluene in the oil by aqueous injection. Transfer of aqueous toluene to the oil phase increased its concentration by up to 10-fold, depending on the aqueous toluene concentration used and the duration of the injection (Gassara et al., 2015). Use of toluene-enriched heavy oil and subsequent injection of nitrate and a toluene-oxidizing, nitrate-reducing enrichment culture gave production of 17.9 ± 1.7% of ROIP (Gassara et al., 2015), whereas in the absence of inoculum and toluene and/or nitrate only 2.5 ± 0.1% of ROIP was produced (Table 3). Gassara et al. (2015) found that a mixture of heptane and toluene was also effective and we have found in the present study that ethylbenzene is as effective as toluene. Note that we mention use of a heptane-toluene mixture in this sentence. Assuming adequate aqueous solubility the choice of the most appropriate low molecular weight hydrocarbon depends on availability, price and regulatory considerations.

Ethylbenzene and toluene are important electron donors for nitrate reduction (Rabus and Widdel, 1995; Chakraborty and Coates, 2004; Lambo et al., 2008; Agrawal et al., 2012; Suri et al., 2017). Toluene is anaerobically metabolized via fumarate addition while ethylbenzene oxidation involves dehydrogenation of the ethyl side chain as initial reactions under denitrifying conditions (Champion et al., 1999). Acetate and benzoate are intermediates of these metabolic pathways. *Thauera* and *Pseudomonas* were the top two taxa in the batch and continuous cultures used here (Figure 2 and Supplementary Table S3). *Thauera* sp. NS1 and *Pseudomonas* sp. NS2, isolated in this study, were able to grow on acetate, benzoate, ethylbenzene and toluene under denitrifying conditions (Figure 4). *Thauera* is a genus of well-studied denitrifying species, which can also use phenylacetate, indole, p-cresol, phenol and other electron donors (Spormann and Widdel, 2000; Mechichi et al., 2002; Fida et al., 2016). Isolate *Thauera* sp. NS1, had 99.0% 16S rRNA gene sequence similarity to *Thauera aromatica* strain S100, which uses benzoate but not toluene or ethylbenzene (Tschech and Fuchs, 1987; Mechichi et al., 2002). Our isolate *Thauera* sp. NS1 uses acetate, benzoate, toluene and ethylbenzene for nitrate reduction (Figure 4). Isolate *Pseudomonas* sp. NS2 also uses acetate, benzoate, toluene and ethylbenzene for nitrate reduction (Figure 4) and had 99.0% similarity to *Pseudomonas stutzeri* strain NBRC 12695. *P. stutzeri* strains use volatile fatty acids (VFA, which are acetate, propionate and butyrate), as well as aromatic compounds like benzoate, toluene, cresols and naphthalenes (Rijn et al., 1996; Arenghi et al., 1999; Lalucat et al., 2006).

Denitrifying ABO-NRB, such as *Thauera* sp. NS1 and *Pseudomonas* sp. NS2, can contribute to MEOR through production of N_2_ and CO_2_ gasses (Supplementary Table S5), which can push oil and water from porous media. The volume of gas formed depends on pressure and temperature, e.g., at 1 atm and 25°C a mole of gas occupies 25 L, whereas at 27.2 atm, as used in our experiments, this is only 0.9 L. Injection of these gasses at very high pressures (10^2^–10^3^ atm) increases their concentration in oil sufficiently to cause viscosity reduction, which is an established method of improving oil production (Holm and Josendal, 1974; Khatib and Earlougher, 1981; Hudgins et al., 1990; Ghasemi and Shadizadeh, 2011). A pressure of 27.2 atm is thus too high to produce oil by gas production, whereas it is too low to cause gas-mediated viscosity reduction. Thus, the possibility that gas production contributed to oil production from our high pressure columns is small.

Injection of low (OD_600_ = 0.090) to high (OD_600_ = 1.769) ABO-NRB biomass concentrations into the columns (Table 3) indicated that oil production increased with the injected biomass concentration both in the absence and presence of nitrate (Figure 5). *Thauera* has previously been found to attach to oil (Kryachko et al., 2012), whereas *Pseudomonas* can attach to hydrocarbon by increasing its cell surface hydrophobicity (Zhang and Miller, 1994; Beal and Betts, 2000; Obuweke et al., 2008; Fernandez et al., 2019). Theoretical calculations, indicated in the text with Supplementary Figure S5, indicate that attachment of ABO-NRB cells (1.3 × 10^9^ cells/cm^3^) to the oil phase can emulsify 1.6 cm^3^ of oil per 17.5 cm^3^ of the aqueous pore volume that was present in our columns (Table 3), when oil emulsion droplets of an average diameter of 250 μm are formed. Although there are quite a few assumptions in these calculations it is interesting that emulsification can in principle produce the 2 cm^3^ of oil per 17.5 cm^3^ of aqueous volume seen in our experiments (Table 3). In reality, the contribution of emulsification is likely less, whereas the largest emulsion droplets may also contribute to through flow diversion. Injection of similar sand-packed columns with sufficient aqueous molasses, glucose or acetate to reduce 80 mM of injected nitrate gave production of 14, 11 and 18% of ROIP, respectively, under low pressure (1 atm) conditions (Gassara et al., 2017). Hence, hydrocarbon- and nitrate-mediated MEOR is similarly effective as carbohydrate- and nitrate-mediated MEOR, which likely involves a different mechanism that does not involve attachment of cells to oil, e.g., through biosurfactants (Okpokwasili and Ibiene, 2006; Youssef et al., 2007; Safdel et al., 2017).

Oil production using pure isolates *Thauera* sp. NS1 and *Pseudomonas* sp. NS2 was somewhat lower than that using ABO-NRB batch and continuous cultures (Table 3), indicating that other taxa in the microbial community also promoted hydrocarbon- and nitrate-mediated MEOR.

In summary, these results indicate that stimulating the microbial communities that are naturally present in low temperature, heavy oil-containing fields by injection of low molecular weight, water-soluble hydrocarbons followed by injection of nitrate and nutrients (e.g., phosphate) is the most promising strategy. Injection of strains with particular metabolic characteristics, as in many applications of carbohydrate- and nitrate-mediated MEOR (Youssef et al., 2007, 2009) is not needed. The injected low molecular weight hydrocarbon is transferred to the oil phase. Attachment to oil and growth of oil-attached bacteria on this low molecular weight hydrocarbon and aqueous nitrate leads to emulsification. The calculations presented indicate that this mechanism can account for the production of residual oil in place observed in this study.

## Data Availability

The datasets generated for this study can be found in Genbank and NCBI, MK085068 and MK085069; SAMN10280305 to SAMN10280325.

## Author Contributions

NS planned and conducted the experiments, collected, analyzed, and interpreted the data, drafted and revised the manuscript. FG helped in conducting the initial experiments. PS helped in setting and operating the high-pressure columns. GV supervised the work through ideas and discussions, critically revised and approved the manuscript to be published.

## Conflict of Interest Statement

The authors declare that the research was conducted in the absence of any commercial or financial relationships that could be construed as a potential conflict of interest.

## Footnotes

1 http://ebg.ucalgary.ca/metaamp

2 http://www.sequencing.ucalgary.ca

3 https://blast.ncbi.nlm.nih.gov/Blast.cgi

4 https://www.ncbi.nlm.nih.gov/nucleotide/
