# Supplementary material for: Microbially Enhanced Oil Recovery by Alkylbenzene-Oxidizing Nitrate-Reducing Bacteria
Source: Front Microbiol. 2019 Jun 18;10:1243. doi: 10.3389/fmicb.2019.01243 (PMC6591262; doi:10.3389/fmicb.2019.01243)
Supplement: Supplementary file 1 [file Table_1.docx]

**Supplementary material**

**Microbially enhanced oil recovery by alkylbenzene-oxidizing nitrate-reducing bacteria**

Navreet Suri^1)*^, Fatma Gassara^1) 2)^, Paul Stanislav^1)^ and Gerrit Voordouw^1)^

^1)^ Petroleum Microbiology Research Group, Department of Biological Sciences, University of Calgary, 2500 University Dr. NW, Calgary, Alberta T2N 1N4, Canada

^2)^ Biopterre, 1642, rue de la Ferme, Saint-Anne-de-la-Pocatière, Quebec G0R 1Z0, Canada

**Table S1:** Components per L of Coleville Synthetic Brine K (CSBK), minimal salts medium used for enrichment of microbial communities. The pH of the medium was adjusted to 7.2-7.5.

• 1.5 g NaCl, 0.05 g KH_2_PO_4_, 0.32 g NH_4_Cl, 0.21 g CaCl_2_·2H_2_O, 0.54 g MgCl_2_·5H_2_O, 0.1 g KCl and 30 ml of 1 M sodium bicarbonate, pH 7

• 1 ml of trace elements (Widdel and Bak, 1992); see below

• 1 ml of tungstate and selenite (Widdel and Bak, 1992); see below

• Trace elements: 993 ml of deionized water, 6.5 ml of HCl (25%, w/v), 1.5 g of FeCl_2_·4H_2_O, 60 mg H_3_BO_3_, 100 mg MnCl_2_·4H_2_O, 120 mg CoCl_2_ 6H_2_O, 70 mg ZnCl_2_, 25 mg NiCl_2_·6H_2_O, 15 mg CuCl_2_·2H_2_O and 25 mg Na_2_MoO_4_·2H_2_O.

• Tungstate and selenite: 1000 ml of deionized water, 8 mg of Na_2_WO_4_, 6 mg of Na_2_SeO_3_·5H_2_O, 400 mg NaOH

**Table S2:** List of 16S rRNA sequences of species of the genus *Thauera* retrieved from the GenBank database of the NCBI and used in the 16S rRNA comparison of **Figure 3**.

| **Species** | **Strain** | **Accession number** | **16S rRNA gene sequence length**  **(bp)** | **Reference** |
| --- | --- | --- | --- | --- |
| *T. terpenica* | 58Eu | AJ005817 | 1484 | Foss and Harder, 1998 |
| *T. humireducens* | SgZ-1 | JQ038037 | 1444 | Yang et al., 2013 |
| *T. aminoaromatica* | S2 | AJ315677 | 1496 | Mechichi et al., 2002 |
| *T. phenylacetica* | B4P | AJ315678 | 1511 | Mechichi et al., 2002 |
| *T. selenatis* | AX39 | NR_026474 | 1491 | Scholten et al., 1999 |
| *T. linaloolentis* | 47Lol | NR_025283 | 1482 | Foss and Harder, 1998 |
| *T. butanivorans* | NBRC 103042 | AB681922 | 1461 | Nakagawa et al., 2011 |
| *T. chlorobenzoica* | 3CB-1 | AF123264 | 1488 | Song et al., 2000 |
| *T. mechernichensis* | TL1 | NR_026473 | 1491 | Scholten et al., 1999 |
| *T. aromatica* | S100 | AJ315681 | 1501 | Mechichi et al., 2002 |
| *T. aromatica* | 2CB2 | AF229881 | 1532 | Song et al., 2000 |
| *T. aromatica* | T1 | U95176 | 1531 | Song et al., 1997 |
| *T. aromatica* | 3CB3 | AF229882 | 1433 | Song et al., 2000 |
| *T. aromatica* | K172 | NR_026153 | 1500 | Anders et al., 1995 |
| *T. aromatica* | LG356 | AJ315680 | 1514 | Mechichi et al., 2002 |
| *T. aromatica* | SP | AJ315679 | 1484 | Mechichi et al., 2002 |
| *T. aromatica* | b66 | EU434574 | 1460 | Li et al., 2009 |

**Table S3:** Microbial community compositions of the produced water 18PW, batch and continuous ABO-NRB cultures (**Table 1 and Figure 1**). Taxa are listed in order of decreasing total fraction with a cutoff of 0.5% of total reads obtained from Illumina sequencing of 16S rRNA amplicons as described in **Figure 2**. Fractions in excess of 1% are shaded.

| **#Taxonomy**  **(Phylum; Class; Order; Family; Genus)** | **18PW** | **B_EN** | **B_N** | **B_EN_EN** | **Day 63** | | | | **Day 23** | | | |  |
| --- | --- | --- | --- | --- | --- | --- | --- | --- | --- | --- | --- | --- | --- |
|  |  |  |  |  | **C_TN (HMN)** | **C_TN** | **C_EN (HMN)** | **C_EN** | **C_TN (HMN)** | **C_TN** | **C_EN (HMN)** | **C_EN** | **B_TN** |
| **# of reads** | **20477** | **26137** | **22238** | **25245** | **30708** | **30150** | **31342** | **28883** | **29585** | **26656** | **30881** | **33924** | **35576** |
| **# of OTU's** | **134** | **94** | **67** | **45** | **96** | **116** | **98** | **107** | **130** | **125** | **116** | **120** | **74** |
| **Chao extrapolated # of OTU's** | **141** | **124** | **91** | **60** | **113** | **150** | **129** | **138** | **148** | **141** | **135** | **137** | **93** |
| **# of taxa** | **88** | **73** | **55** | **38** | **72** | **87** | **73** | **82** | **91** | **88** | **82** | **89** | **62** |
| **Shannon index** | **2.3** | **0.9** | **1.6** | **1.0** | **1.7** | **2.1** | **1.5** | **2.6** | **2.9** | **2.9** | **2.9** | **2.8** | **0.5** |
| Proteobacteria;Betaproteobacteria;Rhodocyclales;  Rhodocyclaceae;Thauera; | 0.03 | 85.27 | 49.10 | 79.85 | 60.27 | 52.30 | 70.16 | 29.00 | 20.31 | 19.47 | 20.33 | 11.07 | 95.33 |
| Proteobacteria;Gammaproteobacteria;Pseudomonadales;  Pseudomonadaceae;Pseudomonas; | 0.02 | 7.15 | 21.65 | 4.48 | 5.29 | 3.10 | 4.38 | 18.45 | 11.00 | 10.95 | 17.34 | 27.03 | 1.43 |
| Bacteroidetes;Sphingobacteriia;Sphingobacteriales;WCHB1-69; | 0.38 | 1.32 | 16.68 | 1.32 | 8.22 | 3.42 | 4.38 | 2.09 | 13.17 | 11.13 | 9.11 | 5.97 | 0.85 |
| Proteobacteria;Betaproteobacteria;Rhodocyclales;  Rhodocyclaceae; | 0.00 | 0.03 | 0.79 | 0.00 | 2.27 | 11.58 | 2.23 | 8.45 | 12.33 | 16.28 | 3.72 | 7.40 | 0.04 |
| Firmicutes;Clostridia;Clostridiales;Peptostreptococcaceae;Acetoanaerobium; | 0.00 | 0.00 | 0.00 | 0.00 | 5.49 | 3.52 | 0.20 | 0.30 | 3.06 | 3.12 | 6.33 | 8.64 | 0.02 |
| Proteobacteria;Deltaproteobacteria;Desulfuromonadales;Desulfuromonadaceae;Desulfuromonas; | 0.00 | 0.02 | 0.00 | 0.00 | 6.91 | 1.78 | 5.23 | 6.27 | 1.35 | 0.12 | 0.85 | 0.92 | 0.00 |
| Spirochaetae;Spirochaetes;Spirochaetales;  Spirochaetaceae;Sphaerochaeta; | 0.02 | 0.16 | 0.05 | 0.02 | 2.26 | 3.93 | 1.33 | 2.38 | 3.54 | 2.21 | 2.56 | 1.90 | 0.14 |
| Euryarchaeota;Methanomicrobia;Methanomicrobiales;  Methanomicrobiaceae;Methanoculleus; | 53.05 | 0.23 | 0.07 | 0.02 | 0.00 | 0.00 | 0.00 | 0.00 | 0.00 | 0.00 | 0.00 | 0.00 | 0.00 |
| Proteobacteria;Betaproteobacteria;Burkholderiales;  Comamonadaceae;Simplicispira; | 0.00 | 0.42 | 0.00 | 0.00 | 0.27 | 0.11 | 0.14 | 0.55 | 2.56 | 7.57 | 3.73 | 2.58 | 0.71 |
| Spirochaetae;Spirochaetes;Spirochaetales;PL-11B10; | 0.02 | 0.00 | 0.00 | 0.00 | 0.01 | 1.83 | 0.50 | 1.12 | 4.61 | 4.00 | 3.54 | 2.41 | 0.16 |
| Proteobacteria;Betaproteobacteria;Rhodocyclales;  Rhodocyclaceae;Denitromonas; | 0.00 | 0.00 | 0.00 | 0.00 | 0.07 | 0.05 | 0.09 | 0.20 | 1.60 | 5.14 | 2.85 | 5.10 | 0.02 |
| Tenericutes;Mollicutes;Acholeplasmatales;  Acholeplasmataceae;Acholeplasma; | 0.01 | 0.00 | 0.14 | 0.00 | 0.00 | 1.18 | 0.02 | 3.93 | 2.41 | 1.19 | 2.33 | 2.86 | 0.03 |
| SHA-109; | 0.00 | 0.00 | 0.00 | 0.00 | 2.12 | 4.27 | 2.31 | 3.60 | 0.03 | 0.01 | 0.00 | 0.02 | 0.00 |
| Bacteroidetes;Bacteroidia;Bacteroidales;  Porphyromonadaceae;Paludibacter; | 0.01 | 0.00 | 0.00 | 0.00 | 0.35 | 1.58 | 1.32 | 2.40 | 1.46 | 1.14 | 1.60 | 1.69 | 0.07 |
| Bacteroidetes;Bacteroidia;Bacteroidales;Rikenellaceae;  vadinBC27-wastewater-sludge-group; | 0.00 | 0.00 | 0.00 | 0.00 | 0.00 | 0.01 | 0.01 | 0.03 | 2.07 | 2.26 | 4.78 | 2.26 | 0.03 |
| Proteobacteria;Alphaproteobacteria;Rhodobacterales;  Rhodobacteraceae; | 0.00 | 0.18 | 2.32 | 0.13 | 0.13 | 0.39 | 0.62 | 3.64 | 0.66 | 0.87 | 0.87 | 2.63 | 0.03 |
| Firmicutes;Clostridia;Clostridiales;Eubacteriaceae;  Acetobacterium; | 0.02 | 0.08 | 0.01 | 0.00 | 0.06 | 0.00 | 0.24 | 0.51 | 0.11 | 0.03 | 4.86 | 4.67 | 0.00 |
| Proteobacteria;Alphaproteobacteria;Rhizobiales;  Rhizobiaceae;Rhizobium; | 0.00 | 0.01 | 0.23 | 0.05 | 0.07 | 0.05 | 0.05 | 0.06 | 1.94 | 1.70 | 3.77 | 1.49 | 0.25 |
| Bacteroidetes;Bacteroidia;  Bacteroidia-Incertae-Sedis;Draconibacteriaceae; | 0.00 | 0.08 | 0.04 | 0.01 | 0.60 | 0.87 | 1.41 | 4.12 | 0.47 | 0.34 | 0.43 | 0.53 | 0.02 |
| Firmicutes;Clostridia;Clostridiales;  Family-XII;Fusibacter; | 0.00 | 0.00 | 0.00 | 0.00 | 0.02 | 0.04 | 0.01 | 0.06 | 0.55 | 0.79 | 2.74 | 3.13 | 0.01 |
| Proteobacteria;Deltaproteobacteria;Desulfuromonadales;Desulfuromonadaceae;Pelobacter; | 0.00 | 0.00 | 0.01 | 0.00 | 0.37 | 0.31 | 0.00 | 0.09 | 2.22 | 1.58 | 1.28 | 1.67 | 0.00 |
| Proteobacteria;Betaproteobacteria;Burkholderiales;  Comamonadaceae; | 0.00 | 0.08 | 0.00 | 0.00 | 0.04 | 0.69 | 0.11 | 0.04 | 4.82 | 0.93 | 0.13 | 0.05 | 0.01 |
| Proteobacteria;Gammaproteobacteria;Xanthomonadales;Xanthomonadaceae; | 0.00 | 0.01 | 0.00 | 0.00 | 1.52 | 1.03 | 0.73 | 0.94 | 0.62 | 1.20 | 0.43 | 0.50 | 0.07 |
| Chlorobi;Ignavibacteria;Ignavibacteriales;BSV26; | 0.00 | 0.00 | 0.00 | 0.00 | 0.38 | 0.93 | 0.88 | 2.29 | 0.43 | 0.05 | 0.62 | 0.15 | 0.00 |

**Table S4:** 16S rRNA gene similarity and sequence distance among *Thauera* species within the microbial communities of batch and continuous cultures of ABO-NRB and *Thauera* sp. NS1 to the known species of *Thauera.* Highlighted numbers are referred to in the text.

| **Species** | **16S rRNA gene sequence similarity (%)** | | | | | | | | | | | | | | | | | | | | | |
| --- | --- | --- | --- | --- | --- | --- | --- | --- | --- | --- | --- | --- | --- | --- | --- | --- | --- | --- | --- | --- | --- | --- |
|  | ***T. terpenica* 58Eu** | ***T. humireducens* SgZ-1** | ***T. aminoaromatica* S2** | ***T. phenylacetica* B4P** | ***T. selenatis* AX39** | ***T. linaloolentis* 47Lol** | ***T. butanivorans* NBRC 103042** | ***T. chlorobenzoica* 3CB-1** | ***T. mechernichensis* TL1** | ***T. aromatica* S100** | ***T. aromatica* 3CB2** | ***T. aromatica* T1** | ***T. aromatica* 3CB3** | ***T. aromatica* K172** | ***T. aromatica* LG356** | ***T. aromatica* SP** | ***T. aromatica* b66** | ***Thauera* B_EN** | ***Thauera* B_TN** | ***Thauera* C_EN(HMN)** | ***Thauera* C_TN(HMN)** | ***Thauera* sp.NS1** |
| ***T. terpenica* 58Eu** | ID | 97.8 | 98.3 | 98.5 | 98.3 | 97.1 | 96.9 | 97.6 | 98.8 | 97.6 | 97.6 | 97.6 | 97.1 | 97.1 | 97.1 | 97.1 | 98.0 | 97.6 | 97.6 | 97.6 | 97.6 | 97.6 |
| ***T. humireducens* SgZ-1** | 0.24 | ID | 99.0 | 99.2 | 99.2 | 97.3 | 97.1 | 97.6 | 98.5 | 97.6 | 97.6 | 97.6 | 97.1 | 97.1 | 97.8 | 97.8 | 98.8 | 97.6 | 97.6 | 97.6 | 97.6 | 97.6 |
| ***T. aminoaromatica* S2** | 0.00 | 0.24 | ID | 99.7 | 99.5 | 98.3 | 98.0 | 98.5 | 99.5 | 98.5 | 98.5 | 98.5 | 98.0 | 98.0 | 98.8 | 98.8 | 99.7 | 98.5 | 98.5 | 98.5 | 98.5 | 98.5 |
| ***T. phenylacetica* B4P** | 0.00 | 0.24 | 0.00 | ID | 99.7 | 98 | 97.8 | 98.3 | 99.2 | 98.3 | 98.3 | 98.3 | 97.8 | 97.8 | 98.5 | 98.5 | 99.5 | 98.3 | 98.3 | 98.3 | 98.3 | 98.3 |
| ***T. selenatis* AX39** | 0.24 | 0.49 | 0.24 | 0.24 | ID | 97.8 | 97.6 | 98.1 | 99.0 | 98.0 | 98.0 | 98.0 | 97.6 | 97.6 | 98.3 | 98.3 | 99.2 | 98.0 | 98.0 | 98.0 | 98.0 | 98.0 |
| ***T. linaloolentis* 47Lol** | 0.24 | 0.49 | 0.24 | 0.24 | 0.00 | ID | 98.8 | 96.9 | 98.3 | 98.8 | 98.8 | 98.8 | 98.3 | 98.3 | 99.0 | 99.0 | 98.0 | 98.8 | 98.8 | 98.8 | 98.8 | 98.8 |
| ***T. butanivorans* NBRC 103042** | 0.98 | 1.23 | 0.98 | 0.98 | 1.23 | 1.23 | ID | 97.1 | 97.6 | 99.0 | 99.0 | 99.0 | 98.5 | 98.5 | 98.8 | 98.8 | 97.8 | 99.0 | 99.0 | 99.0 | 99.0 | 99.0 |
| ***T. chlorobenzoica* 3CB-1** | 1.23 | 1.48 | 1.23 | 1.23 | 1.48 | 1.48 | 0.24 | ID | 98.1 | 98.1 | 98.1 | 98.1 | 97.6 | 97.6 | 97.3 | 97.3 | 98.3 | 98.1 | 98.1 | 98.1 | 98.1 | 98.1 |
| ***T. mechernichensis* TL1** | 1.48 | 1.73 | 1.48 | 1.48 | 1.73 | 1.73 | 0.49 | 0.24 | ID | 98.0 | 98.0 | 98.0 | 97.6 | 97.6 | 98.3 | 98.3 | 99.2 | 98.0 | 98.0 | 98.0 | 98.0 | 98.0 |
| ***T. aromatica* S100** | 0.98 | 1.23 | 0.98 | 0.98 | 1.23 | 1.23 | 0.00 | 0.24 | 0.49 | ID | 100 | 100 | 99.5 | 99.5 | 99.2 | 99.2 | 98.3 | **100** | **100** | **100** | **100** | **100** |
| ***T. aromatica* 3CB2** | 1.48 | 1.73 | 1.48 | 1.48 | 1.73 | 1.73 | 0.49 | 0.74 | 0.98 | 0.49 | ID | 100 | 99.5 | 99.5 | 99.2 | 99.2 | 98.3 | **100** | **100** | **100** | **100** | **100** |
| ***T. aromatica* T1** | 0.00 | 0.24 | 0.00 | 0.00 | 0.24 | 0.24 | 0.98 | 1.23 | 1.48 | 0.98 | 1.48 | ID | 99.5 | 99.5 | 99.2 | 99.2 | 98.3 | **100** | **100** | **100** | **100** | **100** |
| ***T. aromatica* 3CB3** | 0.24 | 0.49 | 0.24 | 0.24 | 0.49 | 0.49 | 1.23 | 1.48 | 1.73 | 1.23 | 1.73 | 0.24 | ID | 99.0 | 98.8 | 98.8 | 97.8 | **99.5** | **99.5** | **99.5** | **99.5** | **99.5** |
| ***T. aromatica* K172** | 0.74 | 0.98 | 0.74 | 0.74 | 0.99 | 0.99 | 1.73 | 1.98 | 2.23 | 1.73 | 1.73 | 0.74 | 0.99 | ID | 98.8 | 98.8 | 97.8 | **99.5** | **99.5** | **99.5** | **99.5** | **99.5** |
| ***T. aromatica* LG356** | 0.49 | 0.74 | 0.49 | 0.49 | 0.74 | 0.74 | 1.48 | 1.73 | 1.98 | 1.48 | 1.98 | 0.49 | 0.74 | 0.74 | ID | 100 | 98.5 | **99.2** | **99.2** | **99.2** | **99.2** | **99.2** |
| ***T. aromatica* SP** | 0.00 | 0.24 | 0.00 | 0.00 | 0.24 | 0.24 | 0.98 | 1.23 | 1.48 | 0.98 | 1.48 | 0.00 | 0.24 | 0.74 | 0.49 | ID | 98.5 | **99.2** | **99.2** | **99.2** | **99.2** | **99.2** |
| ***T.aromatica* b66** | 1.23 | 1.48 | 1.23 | 1.23 | 1.48 | 1.48 | 0.24 | 0.49 | 0.74 | 0.24 | 0.74 | 1.23 | 1.48 | 1.98 | 1.73 | 1.23 | ID | **98.3** | **98.3** | **98.3** | **98.3** | **98.3** |
| ***Thauera* B_EN** | 0.00 | 0.24 | 0.00 | 0.00 | 0.24 | 0.24 | 0.98 | 1.23 | 1.48 | 0.98 | 1.48 | 0.00 | 0.24 | 0.74 | 0.49 | 0.00 | 1.23 | ID | 100 | 100 | 100 | 100 |
| ***Thauera* B_TN** | 0.00 | 0.24 | 0.00 | 0.00 | 0.24 | 0.24 | 0.98 | 1.23 | 1.48 | 0.98 | 1.48 | 0.00 | 0.24 | 0.74 | 0.49 | 0.00 | 1.23 | 0.00 | ID | 100 | 100 | 100 |
| ***Thauera* C_EN(HMN)** | 0.00 | 0.24 | 0.00 | 0.00 | 0.24 | 0.24 | 0.98 | 1.23 | 1.48 | 0.98 | 1.48 | 0.00 | 0.24 | 0.74 | 0.49 | 0.00 | 1.23 | 0.00 | 0.00 | ID | 100 | 100 |
| ***Thauera* C_TN(HMN)** | 2.23 | 2.48 | 2.23 | 2.23 | 2.48 | 2.48 | 1.23 | 0.98 | 1.23 | 1.23 | 0.74 | 2.23 | 2.48 | 2.48 | 2.73 | 2.23 | 1.48 | 2.23 | 2.23 | 2.23 | ID | 100 |
| ***Thauera* sp.NS1** | 1.98 | 2.23 | 1.98 | 1.98 | 2.24 | 2.24 | 0.99 | 0.74 | 0.74 | 0.99 | 1.49 | 1.98 | 2.24 | 2.75 | 2.48 | 1.98 | 1.23 | 1.98 | 1.98 | 1.98 | 1.73 | ID |
|  | **16S rRNA gene sequence distance** | | | | | | | | | | | | | | | | | | | | | |

**Table S5:** Reaction stoichiometries for oxidation of organics with nitrate; all are for 10 mM nitrate because 10 mM nitrate was used in the continuous cultures.

**Table S6:** Summary of oil production from high pressure columns. These contained on average 0.5 PV of heavy MHGC oil and 0.5 PV of aqueous phase. Ethylbenzene (E) or toluene (T) were added to the oil phase, whereas nitrate (N) and ABO-NRB inoculum were injected with the aqueous phase, as indicated. Following 2.5 weeks of incubation flow was resumed and oil production was measured. Additional information is provided in **Table 3**.

| **Column** | **PV**  **(ml)** | **ROIP^1^**  **(ml)** | **Substrates^2^** | **Nitrate reduction**  **(%)^3^** | **Oil produced**  **(ml)** |
| --- | --- | --- | --- | --- | --- |
| 1, 2 | 37.2 ± 0.3 | 19.7 ± 0.5 | T | NA^4^ | 0.5 ± 0.1 |
| 3, 4 | 34.8 ± 0.3 | 14.6 ± 0.8 | N | ND^5^ | 0.8 ± 0.6 |
| 5, 6 | 18.2 ± 1.3 | 7.1 ± 0.1 | EN | 89.3 ± 1.0 | 1.2 ± 0.0 |
| 7 | 36.2 | 20.9 | E | NA^3^ | 1.4 |
| 8 | 36.4 | 22.6 | E | NA^3^ | 1.2 |
| 9 | 35.0 | 16.6 | T | NA^3^ | 1.8 |
| 10 | 35.1 | 15.0 | T | NA^3^ | 2.1 |
| 11 | 35.5 | 21.4 | EN | 96.4 | 2.0 |
| 12 | 35.6 | 19.2 | EN | 87.3 | 1.8 |
| 13 | 35.2 | 16.5 | TN | 61.9 | 2.6 |
| 14 | 34.9 | 14.5 | TN | 56.9 | 3.2 |
| 15-17 | 34.3 ± 3.1 | 16.5 ± 2.3 | TN | 58.2 ± 5.1 | 1.8 ± 0.9 |
| 18-20 | 37.5 ± 0.9 | 19.2 ± 0.7 | TN | 51.9 ± 3.4 | 1.4 ± 0.4 |
| 21-23 | 39.5 ± 1.9 | 20.3 ± 2.6 | TN | 59.0 ± 13.4 | 2.1 ± 1.3 |

^1^ ROIP (ml) = PV (ml) – produced oil (ml)

^2^ Substrates were (E) 9.5 mM of ethylbenzene in the oil phase, (T) 11.2 mM toluene in the oil phase, and/or (N) 80 mM nitrate in the aqueous phase of the columns.

^3^ Nitrate reduction (%) = (nitrite concentration/initial nitrate concentration)*40% + ((initial nitrate concentration - residual nitrate concentration - nitrite concentration)/ initial nitrate concentration)*100%.

^4^ NA = not applicable

^5^ ND = not determined

**Figure S1:** Schematic of continuous cultures. The arrows (**>**) indicate the direction of flow. Samples were taken at the sampling port shown in red.

**Figure S2:** Microscopic images of isolates (A) *Thauera* sp. NS1 and (B) *Pseudomonas* sp. NS2.

**Figure S3:** Concentrations of nitrate (⯁) and nitrite (⯀) in batch cultures with 5% (v/v of total volume of 50 mL) of *Thauera* sp. NS1 and 5% (v/v of total volume of 50 mL) of *Pseudomonas* sp. NS2 with (A) 6.5 mM of aqueous acetate, (B) 1.5 mM of aqueous benzoate, (C) 71.2 mM toluene in 1 mL of HMN phase and (D) 60.6 mM ethylbenzene in 1 mL of HMN phase as electron donors and 10 mM aqueous nitrate as electron acceptor. Additional 5 mM of aqueous acetate (A) and 2 mM of aqueous benzoate (B) was added to the incubations at the indicated times (↓).

**Figure S4:** Differential pressure ΔP (mBar) for high-pressure columns with 0.5 PV of ABO-NRB consortium with 80 mM nitrate. Columns were without (3, 4; ⯁) or with (5, 6; ⯀) 9.5 mM additional ethylbenzene in the oil phase. We describe this in the text as with or without nitrate.

**Figure S5:** Relation of volume of emulsified oil (ml per ml of aqueous phase) and the radius R (µm) of emulsified oil droplets. The arrow represents oil droplets of R=125 µm for which the calculation is done in the text below.

**Emulsification of oil droplets by cell attachment (text explaining Figure S5)**

The columns used were packed with sand particles of 140-200 mesh (200-300 µm). Hence, aqueous flow channels would be blocked if particles of the order of 250 µm (radius R of 125 µm for spherical particles) are generated in the columns, causing diversion of the injected water flow to produce more oil.

Biomass was injected and enhanced by growth on oil-bound ethylbenzene or toluene and aqueous nitrate. For the purpose of this calculation we assume that biomass with an OD_600_=1 was present and that this corresponds to a wet weight of 5 g of biomass/L. We also assume that biomass has a density of 1.3 g/cm^3^ and that individual cells can be approximated as being rectangular with dimensions of 1x1x3 µm. These attach to oil (Kryachko et al., 2012). We assume that attachment is through one of the largest surfaces of 1x3 µm^2^.

With these assumptions the weight of an individual cell is calculated as 3.9x10^-12^ g and the concentration of cells in the aqueous phase as 1.3x10^9^ cells/cm^3^. If all of these bind to the surface of oil droplets with R= 125 µm (surface area of 2x10^5^ µm^2^) then 6.5x10^4^ cells will cover a droplet. Hence, when all 1.3x10^9^ cells/cm^3^ bind to the surface of oil droplets these can emulsify 1.8x10^4^ oil droplets/cm^3^. These have a combined volume of 0.09 cm^3^ of oil per cm^3^ of aqueous pore volume. This corresponds to 1.6 cm^3^ of oil for a column with PV = 35 ml of which half is occupied by water and half is occupied by oil, as in our experiments. The generation of large biomass-covered oil emulsion droplets of 250 µm diameter will block a portion of the aqueous flow channels, causing flow diversion and production of additional oil. Hence, the 2 cm^3^ of oil per column found to be produced in our experiments (**Table 3**) may be produced both by oil emulsification and by blocking of aqueous flow paths by large emulsion droplets and flow diversion. The contribution made by oil emulsification in these calculations is overestimated because not all cells will bind to the oil-water interface and emulsify oil. Also many oil droplets will be smaller and will not contribute to flow diversion. On the other hand, cells may crosslink emulsion droplets causing smaller droplets to move into R>125 µm size range.

Emulsification amplifies the volume of the produced biomass. E.g. if the biomass represented by 1.2x10^9^ cells/cm^3^ would block aqueous flow channels directly by forming 250 µm spherical aggregates, then these would form only 750 aggregates with a combined volume of 0.0038 cm^3^/ cm^3^. This is 24-fold less than the 0.09 cm^3^/cm^3^ obtained by oil emulsification. Hence, emulsification of oil into droplets amplifies the formation of larger sized, flow diverting particles. This volume amplification increases linearly with the average size of the emulsion droplets, because the ratio of volume to area of spheres as a function of R increases by 3/16R (**Figure S5**).

**References**

Anders, H. J., Kaetzke, A., Kampfer, P., Ludwig, W. and Fuchs, G. (1995) Taxonomic position of aromatic-degrading denitrifying *Pseudomonad* strains K 172 and KB 740 and their description as new members of the genera *Thauera,* as *Thauera aromatica* sp. nov., and *Azoarcus,* as *Azoarcus evansii* sp. nov., respectively, members of the beta subclass of the *Proteobacteria.* *Int. J. Syst. Bacteriol*. 45: 327-333.

Foss, S. and Harder, J. (1998) *Thauera linaloolentis* sp. nov. and *Thauera terpenica* sp. nov., Isolated on Oxygen-containing Monoterpenes (Linalool, Menthol, and Eucalyptol) and Nitrate. *System.* & *Appl. Microbiol.* 2: 365-373.

Kryachko, Y., Dong, X., Sensen, C. W. and Voordouw, G. (2012) Compositions of microbial communities associated with oil and water in a mesothermic oil field. *Antonie van Leeuwenhoek.* 101: 493-506. doi: [10.1007/s10482-011-9658-y](https://doi.org/10.1007/s10482-011-9658-y).

Li, D., Yang, M., Hu, J., Zhang, J., Liu, R., Gu, X., Zhang, Y. and Wang, Z. (2009) Antibiotic-resistance profile in environmental bacteria isolated from penicillin production wastewater treatment plant and the receiving river. *Environ. Microbiol.* 11: 1506-1517.

Mechini, T., Stackebrandt, E., Gad’on, N. and Fuchs G. (2002) Phylogenetic and metabolic diversity of bacteria degrading aromatic compounds under denitrifying conditions, and description of *Thauera phenylacetica* sp. nov., *Thauera aminoaromatica* sp. nov., and *Azoarcus buckelii* sp. nov. *Arch. Microbiol.* 178: 26-35. doi: 10. 1007/s00203-002-0422-6.

Nakagawa, Y., Muramatsu, Y., Miyashita, M., Sugimoto, M., Yoshino, M. and Kamakura, Y. (2011) direct submission. <https://www.ncbi.nlm.nih.gov/nuccore/AB681922.1>.

Scholten, E., Lukow, T., Auling, G., Kroppenstedt, R. M., Rainey, F. A. and Diekmann, H. (1999). *Thauera mechernichensis* sp. nov., an aerobic denitrifier from a leachate treatment plant. *Int. J. Syst. Bacteriol.* 49: 1045-1051.

Song, B., Palleroni, N. J. and Haggblom, M. M. (2000) Description of strain 3CB-1, a genomovar of *Thauera aromatica*, capable of degrading 3-chlorobenzoate coupled to nitrate reduction. *Int. J. Syst. Evolution. Microbiol.* 50: 551-558.

Song, B., Young, L. Y. and Palleroni, N. J. (1998) Identification of denitrifier strain T1 as *Thauera aromatica* and proposal for emendation of the genus *Thauera* definition. *Int. J. of Syst. Bacteriol.* 48: 889-894.

Widdel, F. and Bak, F. (1992) Gram-negative mesophilic sulfate-reducing bacteria. *In* A. Balows, H. G. Tru ̈per, M. Dworkin, W. Harder, and K.-H. Schleifer (ed.), The prokaryotes, vol. IV. Springer-Verlag, New York, N.Y. p. 3352–3378.

Yang, G., Zhang, J., Kwon, S., Zhou, S., Han, L., Chen, M., Ma, C. and Zhuang, L. (2013) *Thauera humireducens* sp. nov., a humus-reducing bacterium isolated from a microbial fuel cell. *Int. J. Syst. Evolution. Microbiol.* 63: 873–878.
